# Characterization of an extensive rainbow trout miRNA transcriptome by next generation sequencing

**DOI:** 10.1186/s12864-016-2505-9

**Published:** 2016-03-01

**Authors:** Amelie Juanchich, Philippe Bardou, Olivier Rué, Jean-Charles Gabillard, Christine Gaspin, Julien Bobe, Yann Guiguen

**Affiliations:** INRA, UR1037 LPGP, Campus de Beaulieu, F-35000 Rennes, France; INRA, UMR1388, Plate-forme SIGENAE/GenPhySE, Chemin de Borde Rouge, Auzeville CS 52627, F-31326 Castanet-Tolosan, France; INRA, UR875 Plate-forme GenoToul Bioinfo, Chemin de Borde Rouge, Auzeville CS 52627, F-31326 Castanet-Tolosan, France

**Keywords:** microRNA, Rainbow trout, Repertoire, Tissue expression, Gene regulation

## Abstract

**Background:**

MicroRNAs (miRNAs) have emerged as important post-transcriptional regulators of gene expression in a wide variety of physiological processes. They can control both temporal and spatial gene expression and are believed to regulate 30 to 70 % of the genes. Data are however limited for fish species, with only 9 out of the 30,000 fish species present in miRBase. The aim of the current study was to discover and characterize rainbow trout (*Oncorhynchus mykiss*) miRNAs in a large number of tissues using next-generation sequencing in order to provide an extensive repertoire of rainbow trout miRNAs.

**Results:**

A total of 38 different samples corresponding to 16 different tissues or organs were individually sequenced and analyzed independently in order to identify a large number of miRNAs with high confidence. This led to the identification of 2946 miRNA loci in the rainbow trout genome, including 445 already known miRNAs. Differential expression analysis was performed in order to identify miRNAs exhibiting specific or preferential expression among the 16 analyzed tissues. In most cases, miRNAs exhibit a specific pattern of expression in only a few tissues. The expression data from sRNA sequencing were confirmed by RT-qPCR. In addition, novel miRNAs are described in rainbow trout that had not been previously reported in other species.

**Conclusion:**

This study represents the first characterization of rainbow trout miRNA transcriptome from a wide variety of tissue and sets an extensive repertoire of rainbow trout miRNAs. It provides a starting point for future studies aimed at understanding the roles of miRNAs in major physiological process such as growth, reproduction or adaptation to stress. These rainbow trout miRNAs repertoire provide a novel resource to advance genomic research in salmonid species.

**Electronic supplementary material:**

The online version of this article (doi:10.1186/s12864-016-2505-9) contains supplementary material, which is available to authorized users.

## Background

MicroRNAs (miRNAs) have emerged as important post-transcriptional regulators of gene expression in a wide variety of physiological processes. miRNAs are short endogenous non-coding RNA (ncRNA) present in a wide variety of organisms, including plants, animals, unicellular organisms, and viruses [1]. Mature miRNAs derive from primary transcripts (approximately 1000 bp long) forming hairpins that are cleaved into miRNA precursor (pre-miRNA). One strand of the resulting miRNA duplex is loaded into a miRISC Complex (microRNA Induced Silencing Complex) by Argonaute proteins. Most mature miRNAs are 20–24 nt long while pre-miRNA are 60–80 nt in length. Mainly, the miRISC complex can bind to the 3′UTR of its target mRNA and subsequently lead to degradation or repression of translation [1, 2]. Several examples of non-canonical binding (CDS or 5′UTR) have been reported lately for miRNAs proven to be functional [3]. The role of miRNAs has been studied in several physiological and physiopathological processes. They are involved in key animal developmental processes, such as maternal transcript clearance [4] or axial patterning [5]. Moreover, miRNAs are also involved in disease and 163 pathologies reported in miR2Disease database have been associated with misregulation of 349 miRNA genes or dysfunction of miRNA/mRNA target interaction [6]. In addition, miRNAs can be associated with economical traits. In sheep, a mutation in myostatin 3′-UTR creates a new target site for miRNA and affects muscularity [7]. miRNAs are often expressed in a tissue-enriched manner [8]. They can control both temporal and spatial gene expression and are believed to regulate 30 to 70 % of the protein-coding genes [3].

Before the development of high-throughput sequencing the number of miRNAs discovered by cloning and Sanger sequencing was dramatically underestimated and limited to approximately 100 per species. With the advent of large-scale genomic analyses, even miRNAs with low abundance have been successfully discovered in various species. To date, at least 35,823 mature miRNAs sequence have been discovered, corresponding to a total of 28,645 hairpins sequences in 223 species (miRBase 21, June 2014) [9–11]. Mammals and insects are the most studied species with regards to miRNA discovery but the most recent release (miRBase 21) contains several entries for several fish species such as Atlantic salmon (371 precursors, 498 matures), zebrafish (346 precursors, 350 matures), medaka (168 precursors, 146 matures), common carp (134 precursors, 146 matures), fugu (131 precursors, 108 matures), tetraodon (132 precursors, 109 matures), Atlantic halibut (40 precursors, 37 matures), olive flounder (20 precursors, 38 matures), and channel catfish (281 precursors, 205 matures). All above miRNAs have been discovered by next-generation sequencing. This database is however limited for salmonid species despite the reports of several miRNAs in several tissues of Atlantic salmon [12, 13] or rainbow trout [14, 15] and in rainbow trout eggs [16]. Salmonids are economically and environmentally important species for both wild fisheries and worldwide aquaculture production. Genomic resources for rainbow trout and Atlantic salmon are advanced but our knowledge of salmonid miRNAs repertoire remains incomplete.

The aim of the current study was to discover and characterize rainbow trout (*Oncorhynchus mykiss*) miRNAs in a large number of tissues using next-generation sequencing in order to provide an extensive repertoire of rainbow trout miRNAs. A total of 38 different samples corresponding to 16 different tissues were individually sequenced and analyzed independently in order to identify a large number of miRNAs with high confidence. Differential expression analysis was performed in order to identify miRNAs exhibiting specific or preferential expression among the 16 tissues analyzed. The dataset was also used to discover novel miRNAs that had not been previously reported in other species. This miRNA characterization is a first step to better understand the role of miRNAs in gene regulation in rainbow trout.

## Results and discussion

### Overview of small RNA sequencing

**Fig. 1:**
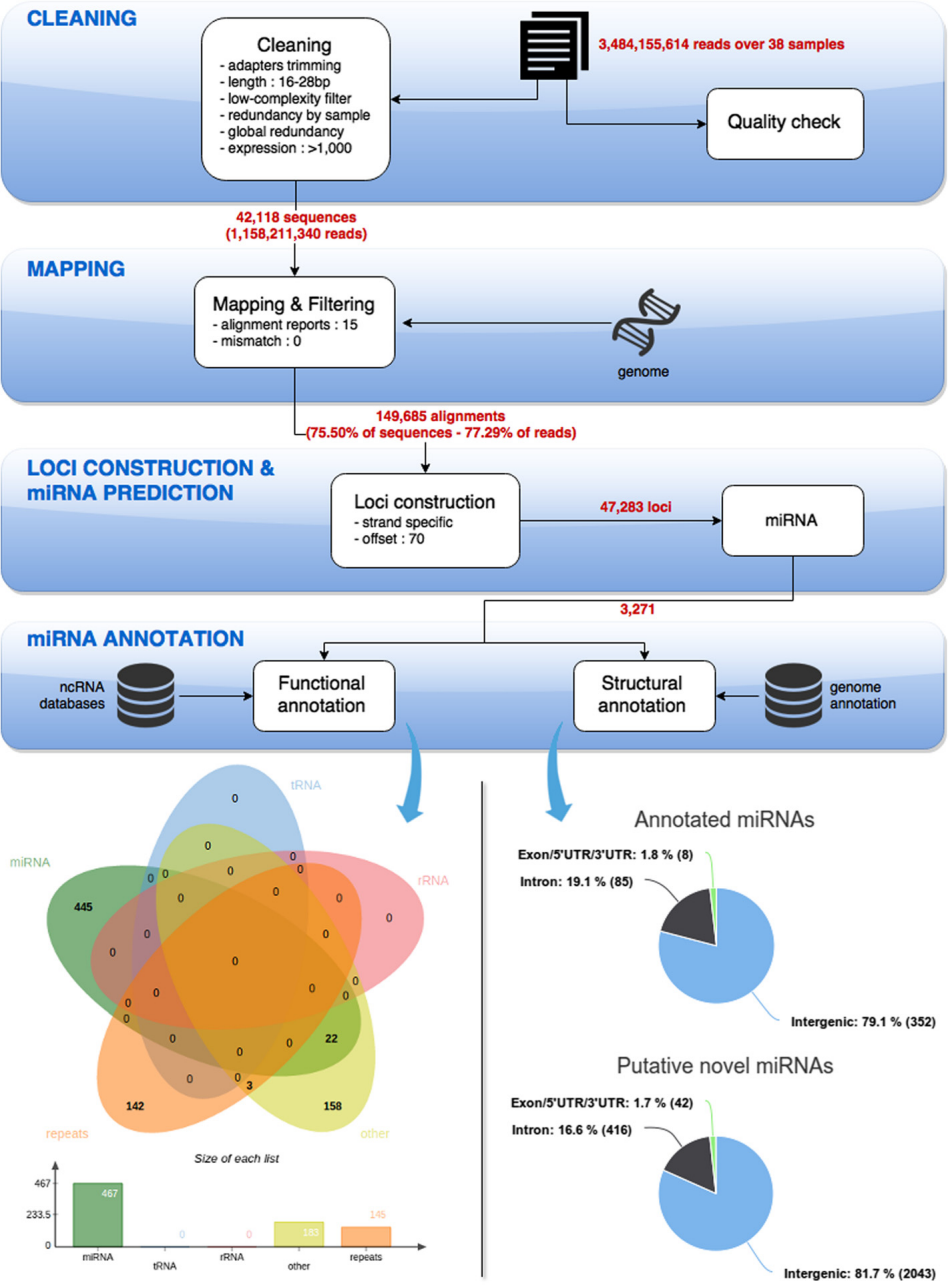
Identification and annotation pipeline of rainbow trout miRNAs using sRNA-sequencing. Fastq files were first cleaned from low quality and adapter sequence. The redundancy was removed from the 38 sample files for the annotation process (*Panel 1*). Unique sequences were then blasted against rRNA, tRNA and RNA database to remove already known RNA sequences that are not miRNA (*Panel 2*). A Blast against miRBase database predicts the already identified miRNAs in other species (*Panel 2*). In parallel, reads were mapped onto the rainbow trout genome to eliminate any contaminant sequences and characterize the miRNA loci. Novel pre-miRNA were also characterized with the specific miRNAs features (*Panel 3*)

**Fig. 2:**
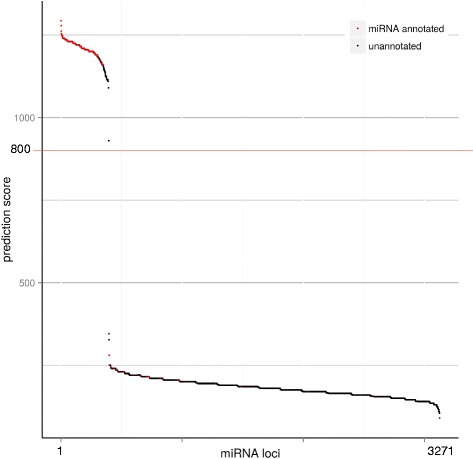
miRNA prediction and genomic localization. The graph represents the distribution of the prediction score for the 3126 miRNAs predicted loci. A clear drop in score numbers is observed around 800. miRNAs annotated loci are in red and unannotated predicted miRNAs loci are in black

Independent small RNA libraries were constructed for 38 samples representing 16 different tissues including gonadal and somatic tissue. A total of 3,484,155,614 reads were generated from the 38 libraries using a HiSeq-1000. The number of reads per sample ranged from 29,326,345 to 126,937,431 (Additional file 1). The processing procedure applied to this dataset is described in Fig. 1. A cleaning procedure was first used that removed adapters sequences and eliminated reads of low complexity. Only the 42,118 non redundant sequences, representing 1,158,211,340 reads with a size in the range of 16–28 nucleotides and a global expression level higher than 1000 were kept for mapping. The mapping procedure allowed to align 75.5 % of the 42,118 non redundant sequences in their full length at 100 % identity onto the rainbow trout genome [17]. A maximum of 15 alignments was reported for each sequence. From the 149,685 alignments that were generated 47,283 loci were built and submitted to the miRNA prediction procedure. 3271 loci were predicted as containing putative pre-miRNA. All the pre-miRNA predicted loci were annotated against ncRNA databases including miRBase 21.0 [11], RFAM 11.0 [18, 19], Silva [20, 21] and GtRNAdb [22]. miRNA from miRBase and RFAM were merged before annotation in order to have a unique miRNA database. Considering the 3271 miRNA predictions, 303 were not considered because they had a perfect match to known repeat elements in rainbow trout (145) or were annotated as other RNA species than miRNA (158), including for example snoRNAs. Three of them were common to repeat elements and ncRNA other than miRNAs. From the 2968 remaining miRNA predictions, 467 were annotated as miRNA. Among them, 22 had multiple annotations in addition to miRNA, including for example snoRNAs and U6 snRNA. 445 predictions were annotated only as miRNA. Regarding the 445 miRNA loci annotation, 332 miRNA loci were annotated in both miRBase and Rfam. 57 miRNA predicted loci were only annotated in miRBase and 56 were only annotated as miRNA in Rfam. Therefore, a total of 2501 miRNA predictions corresponded to new putative miRNA in rainbow trout. The score of predicted miRNA loci, given by the pipeline, ranged from 90 to 1293. The distribution of the score of predicted miRNAs is not linear and a clear drop in score number is observed around 800 (Fig. 2). Loci predicted and annotated as miRNA (with annotation in either miRBase and/or Rfam) have mainly a score higher than 800 (red in Fig. 2). Mis-predicted loci have mainly a score below 250: among the mis-predicted miRNAs loci, only 5 have a score above 800. This threshold of 800 will be further used to select high confidence novel miRNAs among predicted loci. Considering predicted loci annotated as miRNA only (in miRBase, Rfam or both), 352 (79.1 %) are located in intergenic regions, 85 (19.1 %) are in introns, and 8 (1.8 %) are in 5′UTR, 3′UTR or in exons. We observe similar structural annotation percentages for loci predicted as miRNA but not annotated in any database (Fig. 1): 2043 such loci are located in intergenic regions (81.7 %), 416 (16.6 %) are in introns and 42 (1.7 %) are in exons. A complete list of all miRNA loci with structural and functional annotation is provided in Additional file 2.

**Fig. 3:**
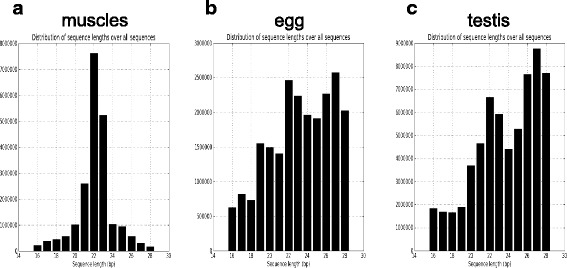
sRNA-seq reads length repartition. **a** The graph represents the read length repartition in a muscle sample (that is representative of all samples except gonadal tissue). **b** The graph represents the read length repartition in the egg sample. **c** The graph represents the read length repartition in the testis stage I sample. All the profiles are represented in Additional file 3

For most samples in the present study, the length distribution of the reads is as expected for a miRNA study, with a peak at 22 nt, which corresponds to the expected miRNA size (Fig. 3A and Additional file 3). For reproductive tissue samples, the distribution is quite different with a first peak at 22 nt and a second peak at 27 nt. The number of reads belonging to the second peak is higher than the one belonging to the first peak. Both ovary (Fig. 3B) and testis (Fig. 3C) exhibit these two peaks in their small RNAs population. The first one is the expected size of miRNAs and the size of the second is consistent with the size of piRNAs (PIWI-Interacting RNAs) that are highly expressed in germ cells [23]. In fact, piRNAs are one of the main classes of non-coding RNAs present in germ cells [23, 24]. Ovarian and testicular samples contain a lot of germ cells and it is therefore not surprising to find this kind of non-coding RNAs in these samples.

**Fig. 4:**
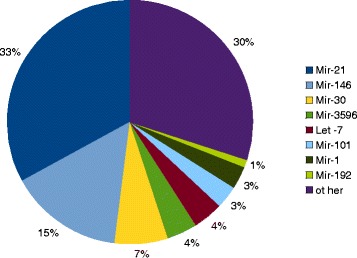
Pie charts of miRNA abundance. Distribution of the miRNA abundance by miRNA family. The top 8 miRNA families are represented (relative expression over 1 %) and represents 70 % of the expressed miRNAs. All the others (miRNA representing individually less than 1 % of the global abundance) represents 30 % of the overall expressed miRNA

### Identification of conserved rainbow trout miRNAs

Many miRNAs have been conserved during evolution and the sequence identity is extremely high even among evolutionary distant species [25–27]. In the present study, we identified rainbow trout miRNAs based on their sequence similarity with other species and used miRBase database (version 21.0) and Rfam database as reference datasets. A total of 445 conserved putative precursors were discovered at unique genome locations that correspond to 123 mature conserved miRNAs. The annotation of miRNAs showed that we have identified 111 evolutionary conserved miRNA families in our dataset (Additional file 2). Several isoforms in sequence and size were found for each miRNA family. This number of identified miRNAs is consistent (i.e in the same range) with miRNA repertoires characterized in other fish species such as salmon [12, 13], zebrafish [28], and medaka [29]. Some miRNAs are present at a unique locus but most of them are present at different positions in the genome (Additional file 2). The large presence of the same miRNA at multiple genomic locations is directly linked to the last round of whole genome duplication (Ss4R) that occurred in salmonids species. First, teleost WGD gave rise to several duplicated miRNA genes located in multiple genomic loci as reported for several teleost fish species. The fourth salmonid-specific event (Ss4R) further bolstered this condition in the genome of salmonid species. As previously shown [17], almost all miRNA loci are retained in duplicated copies in the rainbow trout genome.

### Tissue distribution of rainbow trout miRNAs

Relative abundance of miRNA in the dataset was characterized (Fig. 4). In the global dataset, two miRNAs, miR-21 and miR-146, represent almost half of the reads with 33 and 15 % of the total number of reads in the complete dataset, respectively. Gene expression datasets from several tissues usually exhibit a different pattern: each gene has a small contribution to the total but none of them are overrepresented like it is the case for miRNAs. miR-21 is a well studied miRNA that is involved in different types of cancer [30, 31] and also in the development of heart disease [32]. miR-146 is thought to be a mediator of inflammation and is upregulated by inflammatory factors such as interleukins and tumor necrosis factor-alpha [33]. Eight miRNAs (miR-21, miR-146, miR-30, miR-3596, let-7, miR-101, miR-1 and miR-192) account for more than 1 % each and collectively represent 70 % of the total number of reads. When looking at the miRNA distribution within the different organs (Additional file 4), we found the same miRNAs to be on the top of each list (miR-21, miR-146, miR-143) but also some over-represented miRNA are specific to one organ. For instance, miR-202 accounts for respectively 20 and 10 % of the total reads in testis and ovary, miR-1 accounts for 42 % in muscles and 12 % in heart. A similar distribution was reported in rainbow trout unfertilized eggs [16], with slight differences. let-7 was the most expressed miRNA and accounts for 24 % of the total, followed by miR-21 (18 % of the total). In the present study, we confirm that a small subset of miRNAs is highly expressed and accounts for approximately 70 % of total miRNA counts. This pattern has been observed in salmon fry, but the top expressed miRNAs are not the same. Ssa-miR-181 and ssa-miR-10 account for more than 72 % of the total miRNAs in salmon alevin [13]. The two different studies in salmon do not include as many organs as we have in the present study, so that might be the reason why the top expressed miRNAs are different. In salmon, by looking at a subset of tissue or alevin, they are probably looking at miRNA enrichment in those organs or developmental stages.

**Fig. 5:**
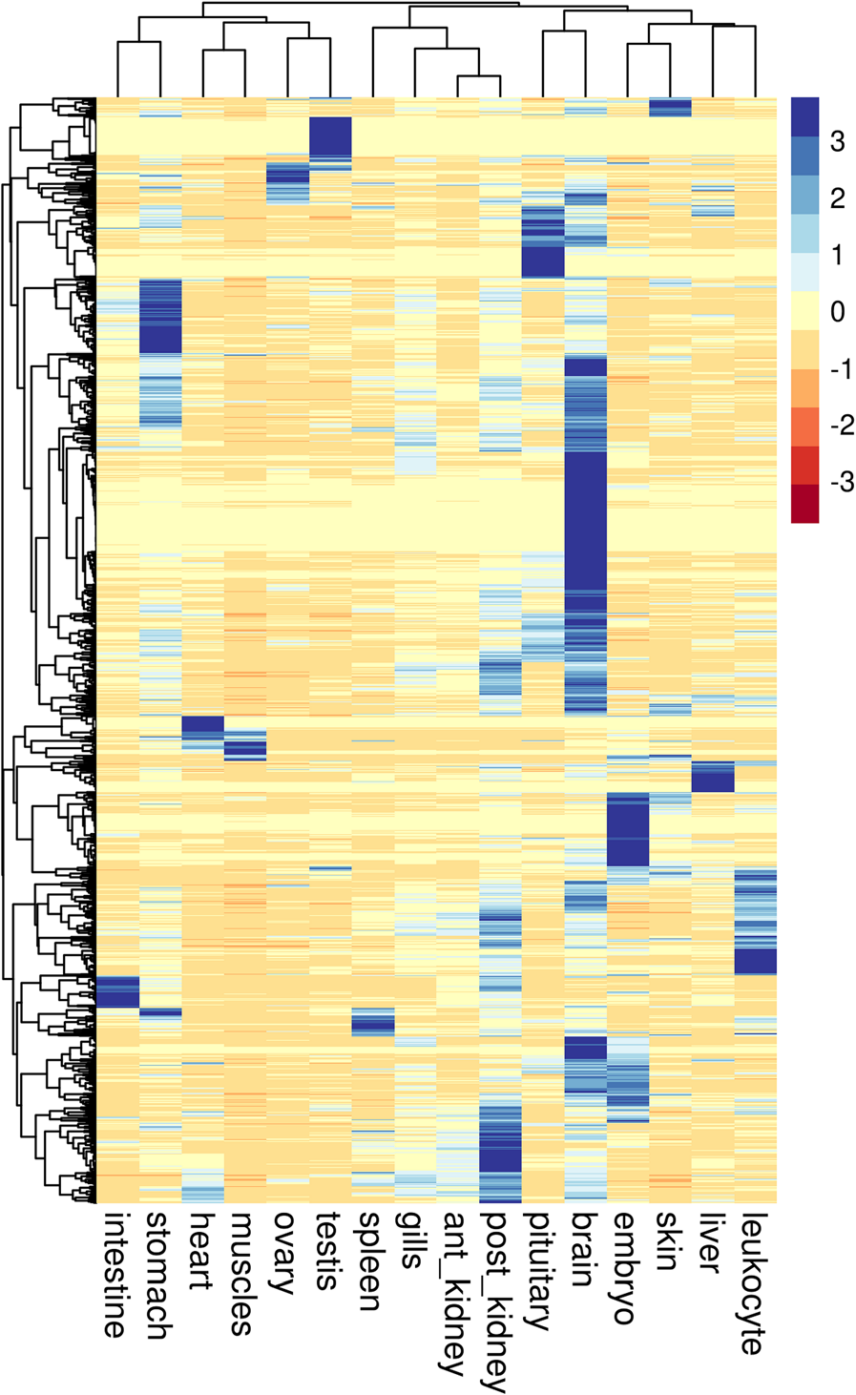
miRNA expression in the 16 tissues. Unsupervised average linkage clustering analysis of miRNA isoforms in rainbow trout tissue. Each row represents a miRNA isoform (total of 1946 rows) and each column a tissue RNA sample. Data were median-centered prior to the clustering analysis. For each miRNA, the expression level within samples is indicated using a color density scale

**Fig. 6:**
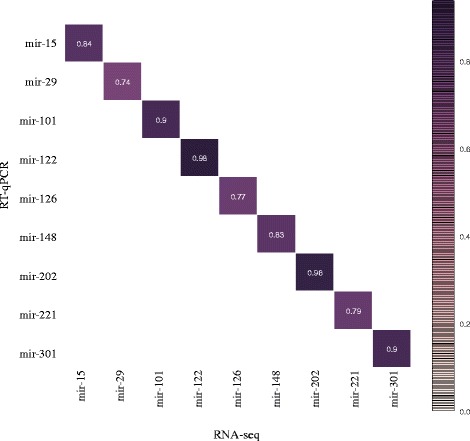
miRNA expression correlation between sRNA-seq and RT-qPCR. Pearson correlations between sRNA-seq and RT-qPCR data were calculated for 9 miRNAs that show diverse expression profiles (tissue enriched, tissue dominant or ubiquitous miRNAs). RNA-seq data are on the y-axis and qPCR are on the x-axis

The strength of our study is that a large number of tissues were sequenced separately. This allows us to perform analysis of miRNA expression among the 16 tissues using unsupervised clustering analysis. The heatMap in Fig. 5 shows the tissue distribution of annotated miRNAs including all isoforms (total of 1946 unique sequences). A large number of miRNAs are clearly overexpressed in a subset of organs/tissues or even only in a single organ/tissue. In mammals, it was demonstrated that miRNAs are expressed in a tissue-dependent manner [7] and rainbow trout miRNAs are clearly following the same rule. This miRNA distribution was validated by RT-qPCR for several selected miRNAs. As shown in Fig. 6, sequencing and RT-qPCR data are highly correlated for the 9 miRNAs studied (Pearson correlation > 0.74). The 9 miRNAs exhibit different patterns of expression (uniquitous vs. organ/tissue enriched expression) and different level of expression. Hereby, we show that sRNA-sequencing can be used for characterizing miRNA repertoire and that expression profiles are confirmed by RT-qPCR. This indicates that the over-representation of some miRNAs does not bias the expression dataset. Another set of samples was used for the RT-qPCR analysis in order to exclude any RNA-seq library preparation bias and to extend the validation to a larger set of samples. Some miRNAs have a ubiquitous expression suggesting a role in common biological processes such as cell proliferation, differentiation or apoptosis. In humans, miR-15 is involved in apoptosis by targeting *BCL2* [34] and miR-29 inhibits cell proliferation and induces cell cycle arrest [35]. Previous studies already reported the expression of those ubiquitous miRNAs in rainbow trout. Salem et al. and Ramachandra et al. [14, 15] identified miRNAs from an RNA pool of nine tissues; muscle, heart, brain, kidney, liver, spleen, intestine, gill, and skin. Most of the ubiquitous miRNAs identified in this study are present in their list. In contrast, some miRNAs have very specific tissue expression pattern. For example (Additional file 5), miR-122 is only expressed in liver and spleen and was shown to be involved in liver metabolism in rainbow trout [36], and miR-202 is only expressed in gonads. The function of miR-202 is not yet understood but it is involved in sex differentiation in chicken [37, 38]. This tissue-enriched expression is mostly conserved from one species to another. For example, miR-202 was found to be gonad-specific in frog [39], Atlantic halibut [40], human, mouse, and rat [7, 41]. Thus, organ/tissue-enriched miRNAs may have a specific role in the organ/tissue and therefore could be key players of evolutionary conserved biological processes.

### Identification of novel rainbow trout miRNAs

**Fig. 7:**
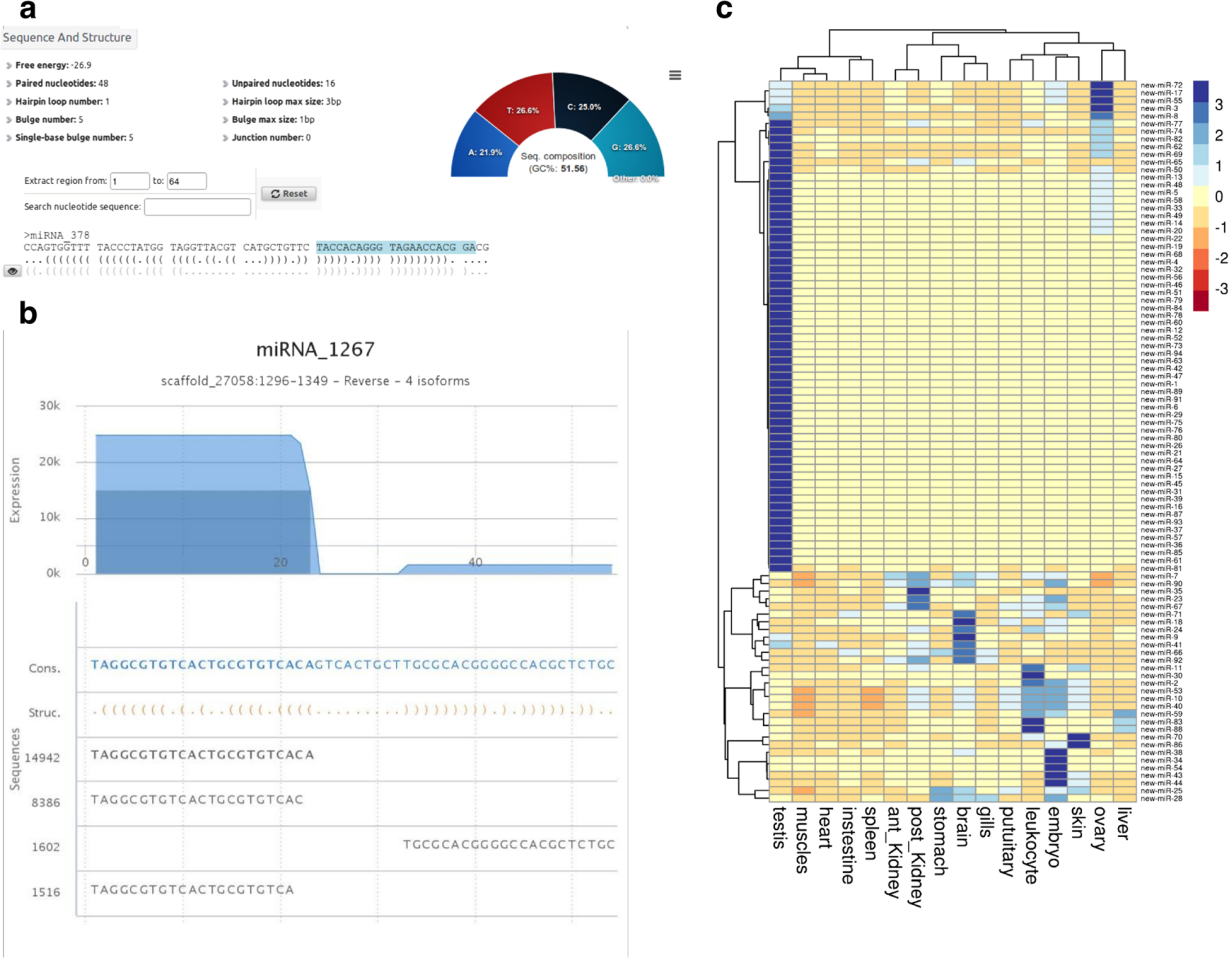
novel miRNAs in rainbow trout: prediction and tissue distribution. **a** The illustration shows sequence and structure statistics of one predicted miRNA precursor as an example. **b** The graph shows, as an example, the 54 nt sequence (putative miRNA precursor) along with reads that aligned to this sequence. The secondary structure of the precursor is shown with the orange parenthesis. **c** Unsupervised average linkage clustering analysis of new miRNA in rainbow trout tissue. Each row represents a new miRNA locus (total of 94 rows) and each column a tissue RNA sample. Data were median-centered prior to the clustering analysis. For each miRNA, the expression level within sample set is indicated using a color density scale

The remaining un-annotated small RNAs mapped to the rainbow trout genome were subjected to novel miRNA prediction. Reads that provided multiple (>15) significant alignments against the rainbow trout genome were not considered as they are most likely interspersed repeats or long tandem repeats. A total of 2501 loci were predicted as miRNA genes without any miRNA annotation. From those 2501 loci, 94 of them had a prediction score greater than 800. As seen previously in Fig. 2, a prediction score of 800 can be used as a threshold for high confidence prediction. Most of the already annoted miRNAs have a prediction score above 800. The above-mentioned 94 loci were therefore classified as high confidence loci while the 2407 remaining loci are classified as low confidence loci. Further analyses were performed for high confidence loci (Additional file 6). Sequencing data were used to build the novel miRNA loci as illustrated in Fig. 7 for one example (panel A and B). Information about the predicted locus are shown in Fig. 7A: it includes structure and sequence data such as free-energy of the hairpin, hairpin sequence and nucleotides distribution. In Fig. 7B, the reads are shown as “stacks” below the precursor sequence and align to both ends of the precursor (both -3p and -5p). The count per base is shown in the top of the B panel. As found in known miRNAs, several isoforms in length are found in the sequencing dataset with a frame shift of 1 or 2 nucleotides in both ends. Expression profiles were also studied for the novel miRNAs (Fig. 7C). As expected most of them display a tissue-specific or tissue-predominant expression profile. This is the case for instance for 59 testis-specific novel miRNAs, 4 ovary-predominant novel miRNAs (new-miR-3, new-miR-17, new-miR-55 and new-miR-72), 2 skin-predominant novel miRNAs (new-miR-70 and new-miR-86). The novel rainbow trout miRNAs may be found in close related species such as salmon but also in any other fish species with the development of sRNA sequencing in economical important species.

### Conservation of identified rainbow trout miRNAs

**Fig. 8:**
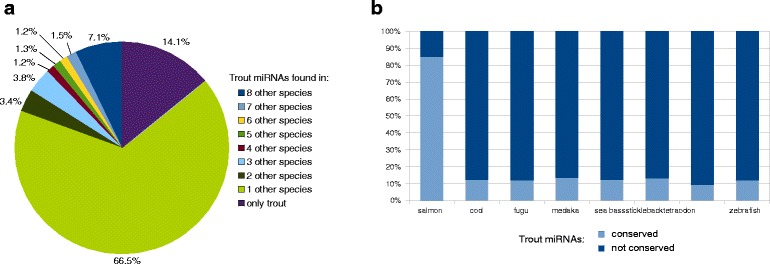
miRNAs conservation among fish species. Conservation analysis of the rainbow trout miRNAs repertoire among 8 fish species: Atlantic salmon, Atlantic cod, zebrafish, medaka, sea bass, stickleback, tetraodon and fugu (**a**) Pie chart representing the percentage of rainbow trout miRNAs that are found among the 8 fish genomes (**b**) Stack histograms representing the conservation of rainbow trout miRNAs in each fish species

miRNAs are known to be conserved among species to a certain extend [42] and therefore we checked whether the identified rainbow trout miRNAs were conserved among fish species. We compared our rainbow trout dataset (all predicted miRNAs in the study, i.e 445 annotated miRNAs plus 2501 putative novel miRNAs) to 8 fish genomes: Atlantic salmon, Atlantic cod, zebrafish, medaka, stickleback, sea bass, tetraodon and fugu and looked for miRNA sequence conservation (Additional file 2). As shown in Fig. 8a, 7 % of the rainbow trout miRNAs are found in all the 8 other selected species, while 67 % of the trout miRNAs are found in only one other species (mainly Atlantic salmon). Those 7 % of miRNAs found in all 8 fish species are common miRNAs also expressed in most vertebrates. One to 4 % of the rainbow trout miRNAs are found in either 2, 3, 4, 5, 6, 7 of the selected species. Interestingly, 14 % of the rainbow trout miRNAs are not found in any of those species, indicating that they might be rainbow trout-specific. It was shown previously in other species that a subset of miRNAs can be species-specific. For instance, 33 miRNAs identified in zebra finch were found to be avian specific and among them, 19 miRNAs were found only in zebra finch [43]. Moreover, as shown in Fig. 8B, most of the rainbow trout miRNAs are found in the Atlantic salmon genome (85 %) while only 9 to 13 % are found in the other species. This could be partially explain by the fact that salmon and rainbow trout are closely related species that share the last round of whole genome duplication (Ss4R) while the 7 other species are more distant from rainbow trout and did not undergo the Ss4R. But, interestingly, the conservation pattern is very similar among these 7 species indicating that after the third round of whole genome duplication (Ts3R), the miRNA repertoire in these fish species is quite stable.

## Conclusions

In conclusion, this study represents the first characterization of rainbow trout miRNA transcriptome from a wide variety of organs/tissues and describes an extensive repertoire of rainbow trout miRNAs. We identified 2946 miRNA loci in the rainbow trout genome including already known miRNAs (445) and putative novel miRNAs (2501 including 94 with high confidence). We also show that differential expression analysis in different tissues is possible using RNA sequencing strategy by confirming the expression data by RT-qPCR with high percentage of correlation. This study provides a starting point for future studies aimed at understanding the roles of miRNAs in major physiological process such as growth, reproduction, nutrition, and adaptation. This rainbow trout miRNAs repertoire provides a novel resource to advance genomic research in salmonids species.

## Methods

### Animals and sample collection

Investigations were conducted according to the guiding principles for the use and care of laboratory animals and in compliance with French and European regulations on animal welfare. Protocols were approved by the Rennes ethical committee for animal research (CREEA). Fish were euthanized using a lethal dose of 2-phenoxyethanol (10 mg/ml of water). All 38 samples (Additional file 1) were immediately frozen in liquid nitrogen and subsequently stored at -80 °C until RNA extraction. Samples from adult fish (heart, stomach, kidney, intestine, pituitary, brain, gills, spleen and skin) were all sampled from the same fish (adult female – 2 years old) in quadruplicates (one for sRNA-seq and the 3 others for RT-qPCR). All others samples were sampled from different fishes, under different experimental or physiological conditions (Additional file 1). Gonies were sampled as previously described in [44].

### RNA extraction

Optimized RNA extraction protocols were used for each tissue as previously described [45]. All samples (except myocytes and myoblastes) were separately homogenized in Tri-reagent (Sigma, St-Louis, USA) at a ratio of 100 mg of tissue per ml of reagent and total RNA was extracted according to manufacturer’s instructions. RNA from myocytes and myoblasts was extracted using the NucleoSpin miRNA kit (Isolation of small and large RNA, Macherey Nagel, Germany) and according to the manufacturer’s instructions. Both long and small RNAs were kept in the same fraction and further referenced as total RNA. Ovarian tissues (ovary and oocytes) were homogenized in Tri-reagent (Sigma, St-Louis, USA) at a ratio of 100 mg of tissue per ml of reagent and total RNA was extracted according to manufacturer’s instructions. Because of high egg yolk content of vitellogenic ovaries and oocytes, all RNA samples were subsequently re-purified using a NucleoSpin miRNA kit (Isolation of small and large RNA, Macherey Nagel, Germany) in order to obtain genomic-grade RNA quality. Both long and small RNAs were kept in the same fraction and further referenced as total RNA. For all samples, RNA integrity was checked using RNA 6000 Nano chip (Agilent).

### Small RNA library and sequencing (Illumina technology)

Small RNA libraries were prepared according to Small RNA v1.5 sample preparation guide (Illumina, January 2010). Briefly, 5 μg of total RNA is used for the small library preparation. Adapters are added at each ends of the small by ligation. First, the ligation is made at the 3′end and then a second ligation is made at the 5′end. v1.5 small RNA 3′ adapter is specifically modified to target miRNAs and other small RNAs that have a 3′ hydroxyl group resulting from enzymatic cleavage by Dicer or other processing enzymes. Small RNAs with both adapters are reverse-transcribed and amplified by PCR. Amplified small RNA libraries are gel purified on a polyacrylamide gel (miRNAs are 89 nt to 96 nt long with both adapters). DNA quality and integrity were checked using DNA-1000 chip (Agilent).

The small library is then hybridized on the flow cell and the libraries’ clustering was performed with the C-Boot cluster machine. The sequencing was done using a HiSeq-1000 sequencer (Illumina).

### Cleaning and redundancy removal

The reads of length 36 nt contain part of the adapter in their 3′ end. We used CutAdapt [46] to remove the part of the adapter occurring at the 3′ end of reads and keep only relevant sequences for further analysis. The trimming was realized on all datasets with options -a ATCTCGTATGCCGTCTTCTGCTTG to remove the Illumina 3′ adapter. Resulting reads in the range 16 to 28 were obtained by setting parameters -m 16 and -M 28. This allowed us to keep reads in an enlarged expected range of miRNA size. Sequences containing ‘N’ and those of low complexity (less than 3 different nucleotides) were removed. Redundancy was then removed by pooling together all datasets and keeping only unique sequences. Finally sequences for which the number of occurrence was lower than 1000 were removed from the datasets. For each unique sequence, its occurrences were summed up and conserved for the dataset of all pooled libraries and for each independent library dataset.

### Identification and annotation of miRNA loci

Analysis of unique sequences led to the identification of several known and novel small ncRNA families, including ribosomal RNA (rRNA), small nucleolar RNA (snoRNA), transfer RNA (tRNA) and many others. Unique cleaned sequences were aligned with Bowtie2 against the available rainbow trout scaffolded sequences [17]. At most 15 hits of exact match were reported for each unique sequence. Loci were built by considering regions on the same strand separated by at most 70 nt. Loci were submitted to the miRNA prediction procedure that considers the expected pre-miRNA stem-loop structure, the size of the pre-miRNA sequence, the size of pre-miRNA loops (bulges, internal loops, stem loop), the size of the most represented sequence (20–24 nt) and its alignment against the stem of the pre-miRNA and the pre-miRNA expected expression profile. A score is assigned to each predicted pre-miRNA locus considering previous characteristics. Each predicted pre-miRNA locus is then submitted to the annotation procedure by aligning it against known ncRNA databases with Blast + [47] allowing mismatches. We selected SILVA [21], GtRNAdb [22], miRBase [11] and Rfam [19] to annotate sequences matching respectively known SSU and LSU rRNA, tRNA, miRNA and ncRNA from other families (Additional file 3). All predicted loci were mapped (Bowtie2 [48], no mismatch) against a repeat elements database [17] to remove transposons sequences from the putative predicted miRNA loci.

Conservation analysis was performed by blasting the identified rainbow trout miRNAs dataset against 8 fish genomes: Atlantic salmon (ICSASG_v1), Atlantic cod (gadMor1), zebrafish (Zv9), medaka (HdrR), seabass, stickleback (BROAD S1), tetraodon (TETRADON 8.0) and fugu (FUGU 4.0). Number of hits and eventual mismatches on the genome were recorded (Additional file 2).

### miRNA reverse transcription and qPCR analysis

Reverse transcription and QPCR were performed as previously described with minor modifications [43]. Briefly, total RNA (500 ng) was reverse transcribed using NCode™ VILO™ miRNA cDNA synthesis kit (Invitrogen, Cergy Pontoise, France) according to the manufacturer’s instructions. Real-time PCR was performed using a Step One Plus thermocycler (Applied Biosystems, Foster City, USA). Reverse transcription products were diluted to 1/100 and 4 μl were used for each real-time PCR reaction. Duplicates were run for each RT product. Real-time PCR was performed using a real-time PCR kit provided with a SYBR Green fluorophore (Fast SYBR Green Master Mix kit, Applied Biosystems) according to the manufacturer’s instructions with 100 nM of each primer (Additional file 7) for miRNAs. After a 30-s incubation step at 95 °C, amplification was performed using the following cycle: 95 °C, 3 s; 60 °C, 30 s; 40 times. The relative abundance of target cDNA within sample set was calculated from a serially diluted cDNA pool using the Step One Plus software. After amplification, a fusion curve was obtained in order to ensure that a single PCR product had been generated using the following protocol: 1 s holding followed by a 0.5 °C increase, from 60 to 95 °C. QPCR signal was normalized using 18S expression which is stable when comparing different tissues.

### Analysis of tissue distribution

A hierarchical clustering was performed to characterize the miRNAs tissue distribution. The analysis was restricted to 16 different and independent tissues in order to characterize any differences between tissues and not between physiological conditions. The aim of the study was to characterize the rainbow trout miRNA repertoire but not to study any differences between several physiological conditions. We sequenced a large number of samples in order to get the most complete repertoire. The 16 chosen tissues are in bold in Additional file 1.

### Availability of supporting data

All the sequencing data are deposited in SRA under the Bioproject accession number PRJNA227065 (http://www.ncbi.nlm.nih.gov/sra/?term=PRJNA227065). A website located at “ngspipelines.toulouse.inra.fr:9064” allows to explore all predicted miRNA providing complete information including functional and structural annotation, locus view and expression visualization by library (Additional file 8).

## Additional files

Additional file 1: Samples list and read counts. The list of the 38 samples that have been used in the study. The samples used for the tissue expression analysis are in bold. (PPT 91 kb) Additional file 2: List of all predicted miRNA loci in the rainbow trout genome with functional, structural and conservation annotation. The list of the 3270 predicted miRNA loci that have been discovered in the study. For each loci, the file gives information about basic information (miRNA id, chromosome location, start and stop of the pre-miR, strand, expresion information, prediction score, pre-miRNA sequence, miRNA sequence, pre-miRNA length and miRNA length), structural annotation (genomic position regarding protein coding genes with sRNA browse annotation or intron annotation), functional annotation for both the pre-miRNA and the mature miRNA (mirbase, Rfam, tRNAs, rRNA or repeat elements annotation) and conservation annotation (conservation of rainbow trout loci in 8 fish species, nm = number of mismatch). (XLSX 1068 kb) Additional file 3: sRNA-seq reads length repartition in the 38 samples. Each panel represents the read length distribution of the 38 RNA samples. The box highlights the gonadal tissues. Each numbers are in reference to the corresponding sample in additional file 1. Highlighted samples (black box with numbers 2, 26, 27, 28 and 29) are gonadal samples. (PPT 727 kb) Additional file 4: top3 most expressed miRNA in each sample. Table showing the top 3 expressed miRNAs in each of the 16 samples with associated percentage. (PPT 59 kb) Additional file 5: qPCR validation of 9 miRNAs. Each panel represents expression of a miRNA in a set of tissues. Nine miRNAs were checked by qPCR: miR-15, miR-29, miR-101, miR-122, miR-126, miR-148, miR-202, miR-221 and miR-301. qPCR expression of each miRNA was normalized using 18S expression. (PPT 112 kb) Additional file 6: List of novel miRNA in the rainbow trout. The list of the 94 predicted new miRNA loci that have been discovered with high confidence in the study. For each loci, the file gives information about: name, miRNA locus ID (same name as in Additional file 4), chromosome location, start and stop of the pre-miR, strand, length of the pre-miR, prediction score, DNA sequence of the pre-miR. (XLSX 13 kb) Additional file 7: List of primers used in this study. (XLSX 4 kb) Additional file 8: Webpage available for each miRNAs. All 3271 loci have a webpage describing miRNA features, structure and reporting the different isoforms produced by each loci. (PDF 490 kb)

## Abbreviations

cDNA: complementary DesoxyriboNucleic acid
miRNA: microRNA
ncRNAs: non-coding RNAs
RNA: RuboNucleic acid
rRNA: ribosomal RNA
RT-qPCR: real-time quantitative polymerase chain reaction
tRNA: transfert RNA
