# Supplementary figures and images for: Characterization of an extensive rainbow trout miRNA transcriptome by next generation sequencing
Source: BMC Genomics. 2016 Mar 1;17:164. doi: 10.1186/s12864-016-2505-9 (PMC4774146; doi:10.1186/s12864-016-2505-9)

## Slide 1
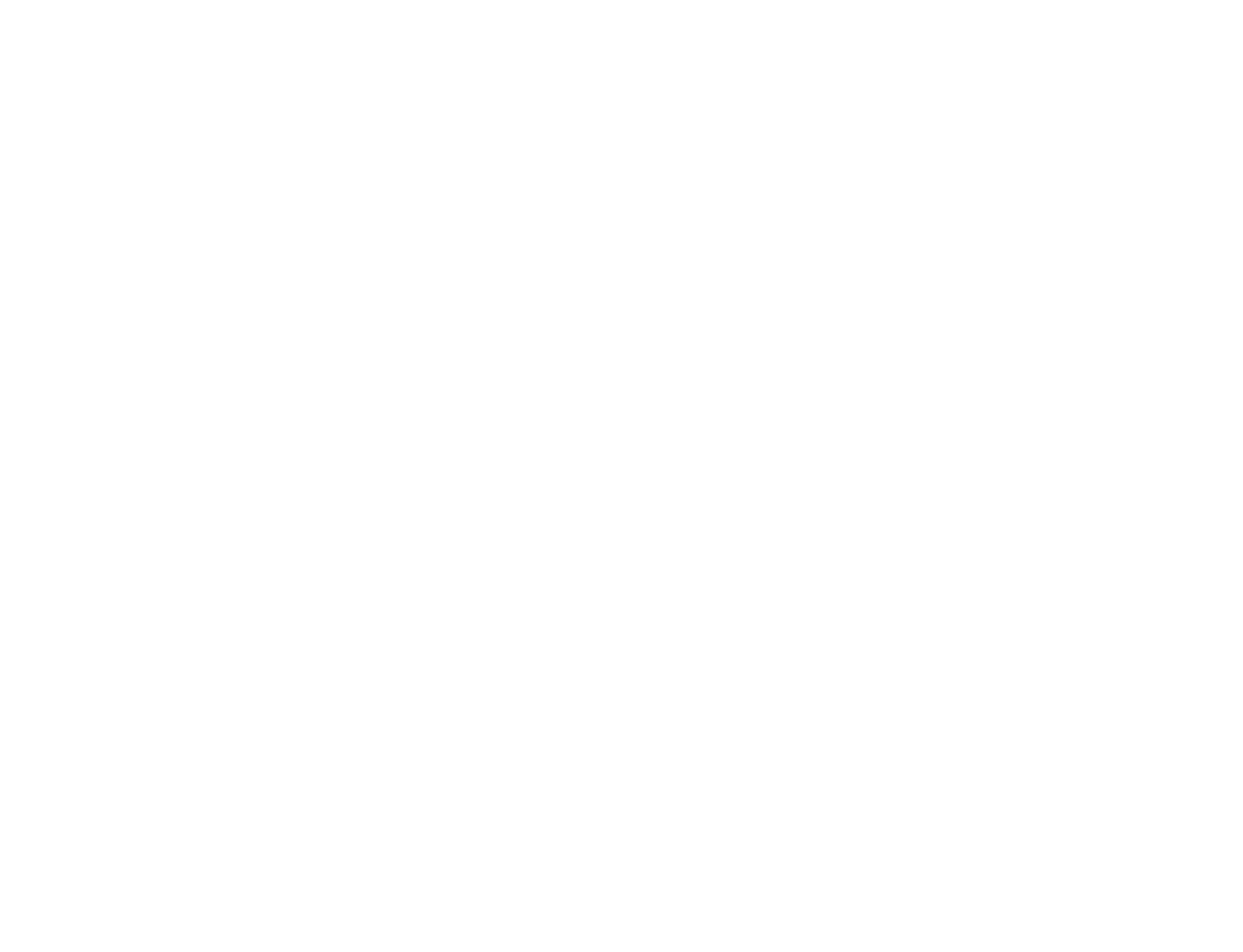

Supplement: Additional file 1: — Samples list and read counts. The list of the 38 samples that have been used in the study. The samples used for the tissue expression analysis are in bold. (PPT 91 kb) [file 12864_2016_2505_MOESM1_ESM.ppt]

## Slide 1
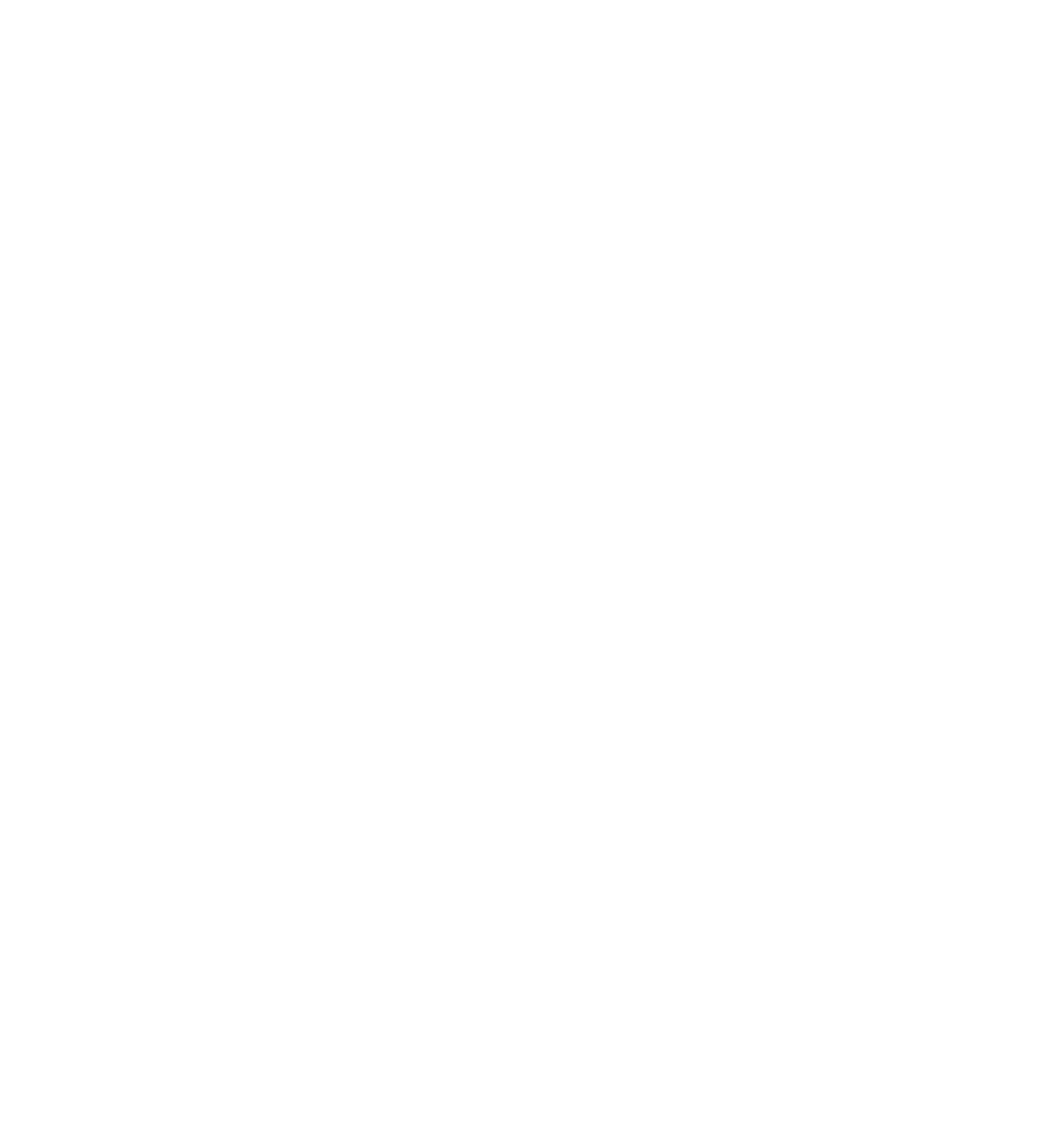

Supplement: Additional file 3: — sRNA-seq reads length repartition in the 38 samples. Each panel represents the read length distribution of the 38 RNA samples. The box highlights the gonadal tissues. Each numbers are in reference to the corresponding sample in additional file 1. Highlighted samples (black box with numbers 2, 26, 27, 28 and 29) are gonadal samples. (PPT 727 kb) [file 12864_2016_2505_MOESM3_ESM.ppt]

## Slide 1
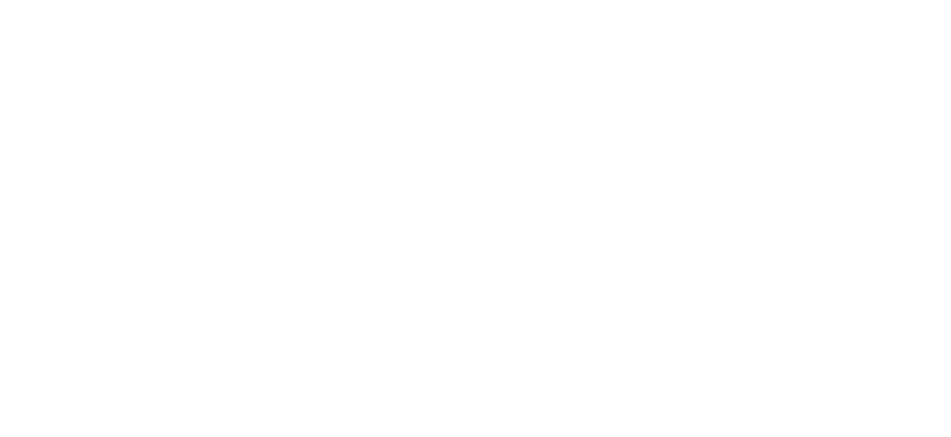

Supplement: Additional file 4: — top3 most expressed miRNA in each sample. Table showing the top 3 expressed miRNAs in each of the 16 samples with associated percentage. (PPT 59 kb) [file 12864_2016_2505_MOESM4_ESM.ppt]

## Slide 1
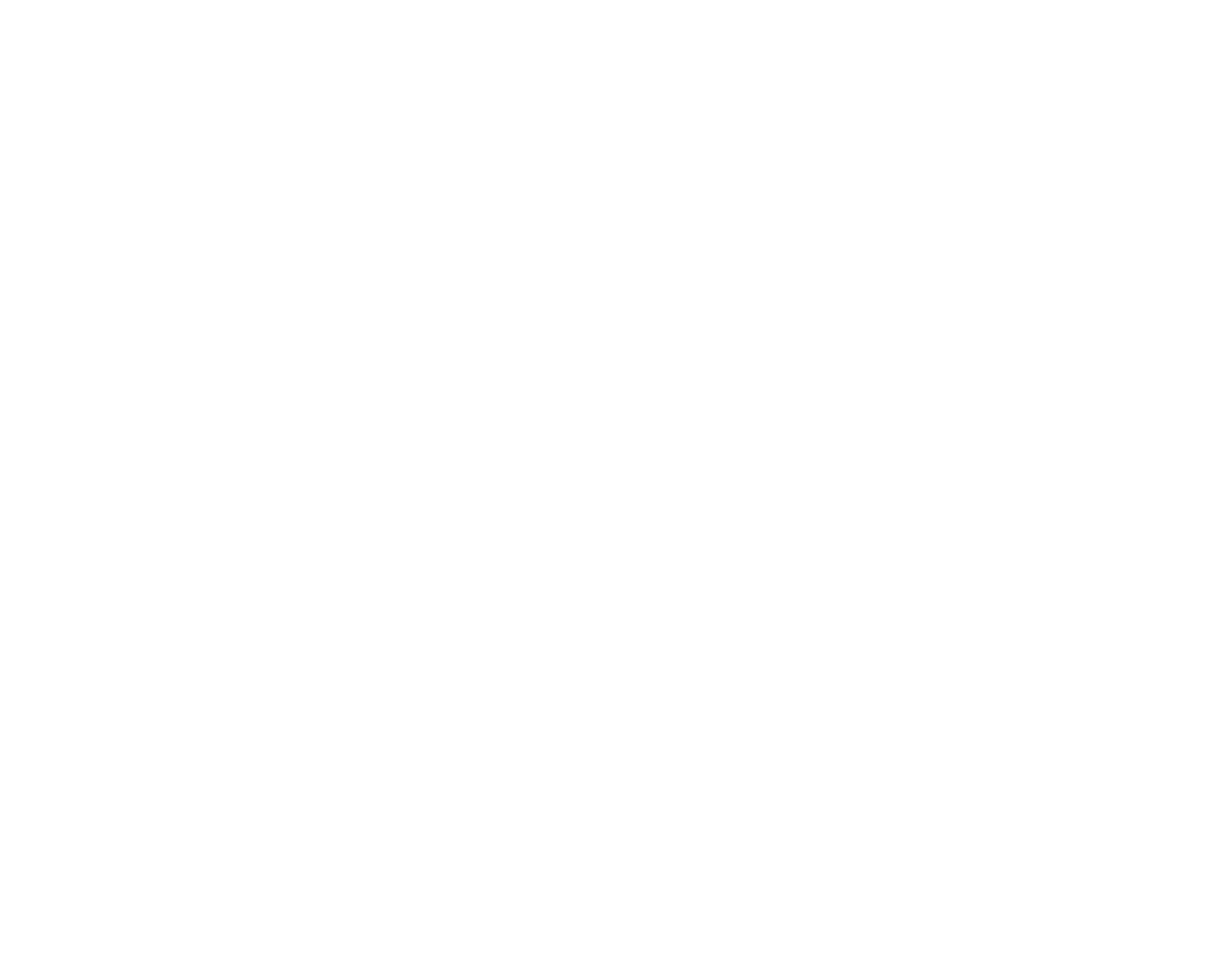

Supplement: Additional file 5: — qPCR validation of 9 miRNAs. Each panel represents expression of a miRNA in a set of tissues. Nine miRNAs were checked by qPCR: miR-15, miR-29, miR-101, miR-122, miR-126, miR-148, miR-202, miR-221 and miR-301. qPCR expression of each miRNA was normalized using 18S expression. (PPT 112 kb) [file 12864_2016_2505_MOESM5_ESM.ppt]
