# Supplementary material for: Characterization of an extensive rainbow trout miRNA transcriptome by next generation sequencing
Source: BMC Genomics. 2016 Mar 1;17:164. doi: 10.1186/s12864-016-2505-9 (PMC4774146; doi:10.1186/s12864-016-2505-9)
Supplement: Additional file 8: — Webpage available for each miRNAs. All 3271 loci have a webpage describing miRNA features, structure and reporting the different isoforms produced by each loci. (PDF 490 kb) [file 12864_2016_2505_MOESM8_ESM.pdf]

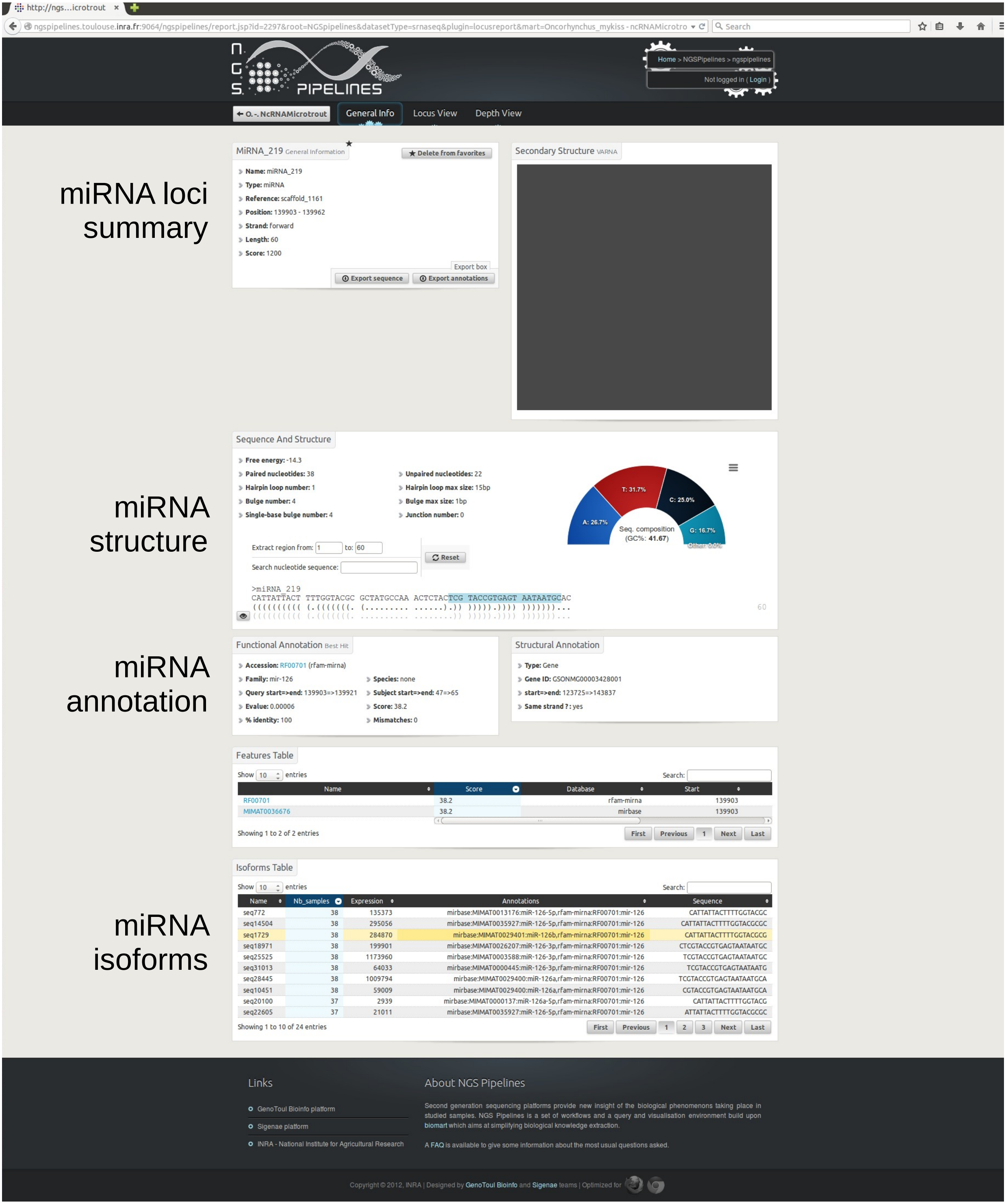
miRNA loci summary
miRNA structure
miRNA annotation
miRNA isoforms
http://ngs...icrotrout
ngspipelines.toulouse.inra.fr:9064/ngspipelines/report.jsp?id=2297&root=NGSpipelines&datasetType=srnaseq&plugin=locusreport&mart=Oncorhynchus_mykiss - ncRNAMicrotro
Search
NGS PIPELINES
Home > NGSPipelines > ngspipelines
Not logged in (Login)
O. -. NcRNAMicrotrout
General Info
Locus View
Depth View
MiRNA_219 General Information
Delete from favorites
Name: miRNA_219
Type: miRNA
Reference: scaffold_1161
Position: 139903 - 139962
Strand: forward
Length: 60
Score: 1200
Export box
Export sequence
Export annotations
Secondary Structure VARNA
Sequence And Structure
Free energy: -14.3
Paired nucleotides: 38
Unpaired nucleotides: 22
Hairpin loop number: 1
Hairpin loop max size: 15bp
Bulge number: 4
Bulge max size: 1bp
Single-base bulge number: 4
Junction number: 0
T: 31.7%
C: 25.0%
A: 26.7%
G: 16.7%
Other: 0.0%
Seq. composition
(GC%: 41.67)
Extract region from: 1 to: 60
Reset
Search nucleotide sequence:
>miRNA_219
CATTATTACT TTTGGTACGC GCTATGCCAA ACTCTACTCG TACCGTGAGT AATAATGCAC
(((((((((((.(((((((. (......... ......).)))))))).))))))))))...
(((((((((((.(((((((. ....)))))))).))))))))))...
60
Functional Annotation Best Hit
Accession: RF00701 (rfam-mirna)
Family: mir-126
Species: none
Query start=>end: 139903=>139921
Subject start=>end: 47=>65
Evalue: 0.00006
Score: 38.2
% identity: 100
Mismatches: 0
Structural Annotation
Type: Gene
Gene ID: GSONMG00003428001
start=>end: 123725=>143837
Same strand ? : yes
Features Table
Show 10 entries
Search:
Name | Score | Database | Start
RF00701 | 38.2 | rfam-mirna | 139903
MIMAT0036676 | 38.2 | mirbase | 139903
Showing 1 to 2 of 2 entries
First Previous 1 Next Last
Isoforms Table
Show 10 entries
Search:
Name | Nb_samples | Expression | Annotations | Sequence
seq772 | 38 | 135373 | mirbase:MIMAT0013176:miR-126-5p,rfam-mirna:RF00701:mir-126 | CATTATTACTTTTGGTACGC
seq14504 | 38 | 295056 | mirbase:MIMAT0035927:miR-126-5p,rfam-mirna:RF00701:mir-126 | CATTATTACTTTTGGTACGCGC
seq1729 | 38 | 284870 | mirbase:MIMAT0029401:miR-126b,rfam-mirna:RF00701:mir-126 | CATTATTACTTTTGGTACGCG
seq18971 | 38 | 199901 | mirbase:MIMAT0026207:miR-126-3p,rfam-mirna:RF00701:mir-126 | CTCGTACCGTGAGTAATAATGC
seq25525 | 38 | 1173960 | mirbase:MIMAT0003588:miR-126-3p,rfam-mirna:RF00701:mir-126 | TCGTACCGTGAGTAATAATGC
seq31013 | 38 | 64033 | mirbase:MIMAT0000445:miR-126-3p,rfam-mirna:RF00701:mir-126 | TCGTACCGTGAGTAATAATG
seq28445 | 38 | 1009794 | mirbase:MIMAT0029400:miR-126a,rfam-mirna:RF00701:mir-126 | TCGTACCGTGAGTAATAATGCA
seq10451 | 38 | 59009 | mirbase:MIMAT0029400:miR-126a,rfam-mirna:RF00701:mir-126 | CGTACCGTGAGTAATAATGCA
seq20100 | 37 | 2939 | mirbase:MIMAT0000137:miR-126a-5p,rfam-mirna:RF00701:mir-126 | CATTATTACTTTTGGTACG
seq22605 | 37 | 21011 | mirbase:MIMAT0035927:miR-126-5p,rfam-mirna:RF00701:mir-126 | ATTATTACTTTTGGTACGCGC
Showing 1 to 10 of 24 entries
First Previous 1 2 3 Next Last
Links
GenoToul Bioinfo platform
Sigenae platform
INRA - National Institute for Agricultural Research
About NGS Pipelines
Second generation sequencing platforms provide new insight of the biological phenomenons taking place in studied samples. NGS Pipelines is a set of workflows and a query and visualisation environment build upon biomart which aims at simplifying biological knowledge extraction.
A FAQ is available to give some information about the most usual questions asked.
Copyright © 2012, INRA | Designed by GenoToul Bioinfo and Sigenae teams | Optimized for
